# Functional differences between neurochemically defined populations of inhibitory interneurons in the rat spinal dorsal horn^☆^

**DOI:** 10.1016/j.pain.2013.05.001

**Published:** 2013-12

**Authors:** Erika Polgár, Thomas C.P. Sardella, Sheena Y.X. Tiong, Samantha Locke, Masahiko Watanabe, Andrew J. Todd

**Affiliations:** a Spinal Cord Group, Institute of Neuroscience and Psychology, University of Glasgow, Glasgow G12 8QQ, United Kingdom; b Department of Anatomy, Hokkaido University School of Medicine, Sapporo 060-8638, Japan

**Keywords:** Galanin, Neuronal nitric oxide synthase, Neuropeptide Y, Pain, Parvalbumin, Somatostatin receptor 2A

## Abstract

In order to understand how nociceptive information is processed in the spinal dorsal horn we need to unravel the complex synaptic circuits involving interneurons, which constitute the vast majority of the neurons in laminae I–III. The main limitation has been the difficulty in defining functional populations among these cells. We have recently identified 4 non-overlapping classes of inhibitory interneuron, defined by expression of galanin, neuropeptide Y (NPY), neuronal nitric oxide synthase (nNOS) and parvalbumin, in the rat spinal cord. In this study we demonstrate that these form distinct functional populations that differ in terms of sst_2A_ receptor expression and in their responses to painful stimulation. The sst_2A_ receptor was expressed by nearly all of the nNOS- and galanin-containing inhibitory interneurons but by few of those with NPY and none of the parvalbumin cells. Many galanin- and NPY-containing cells exhibited phosphorylated extracellular signal-regulated kinases (pERK) after mechanical, thermal or chemical noxious stimuli, but very few nNOS-containing cells expressed pERK after any of these stimuli. However, many nNOS-positive inhibitory interneurons up-regulated Fos after noxious thermal stimulation or injection of formalin, but not after capsaicin injection. Parvalbumin cells did not express either activity-dependent marker following any of these stimuli. These results suggest that interneurons belonging to the NPY, nNOS and galanin populations are involved in attenuating pain, and for NPY and nNOS cells this is likely to result from direct inhibition of nociceptive projection neurons. They also suggest that the nociceptive inputs to the nNOS cells differ from those to the galanin and NPY populations.

## Summary

Neurochemically defined populations of inhibitory interneurons in the superficial dorsal horn of the spinal cord differ in their receptor expression pattern and responses to noxious stimuli.

## 1 Introduction

The great majority of neurons in laminae I–III of the dorsal horn are interneurons with axons that arborize locally, and these play a major part in the neuronal circuits that process sensory inputs, including those perceived as pain [2,11,37,52,60,66,77,80]. Our understanding of the organisation of these circuits remains limited, mainly as a result of the difficulty of defining functional populations among the interneurons [11,66,80]. Inhibitory interneurons that use GABA and/or glycine constitute 25–40% of the neurons in laminae I–III in the rat [45]. Several roles have been suggested for these cells, including prevention of different types of pain [52,60,77] and suppression of itch [50]. In addition, loss of function of the inhibitory interneurons (eg, as a result of the decreased synthesis of GABA or reduction of its postsynaptic action) may contribute to neuropathic pain [6,7,39,44,56]. Previous attempts to classify dorsal horn interneurons on the basis of morphological and electrophysiological criteria have met with limited success. Although some inhibitory interneurons in lamina II have been identified as islet or central cells [12,15,16,32,36,67,79,81], many do not belong to these classes and are morphologically diverse [16,36,79]. Even less is known about inhibitory interneurons in laminae I and III.

Neurochemistry provides an alternative approach for classifying these cells, and we have identified 4 non-overlapping populations of inhibitory interneurons in laminae I–III of the rat, based on expression of neuropeptide Y (NPY), galanin, neuronal nitric oxide synthase (nNOS) or parvalbumin [28,46,54,64]. Between them, these account for at least half of the inhibitory interneurons in laminae I–II [54], and it has been demonstrated that there are differences in the postsynaptic targets of their axons [21,46,47,49,54]. Developmental studies also indicate a different lineage for NPY- and galanin-containing cells in the mouse [4].

The somatostatin receptor sst_2A_, which is present at high levels in the superficial dorsal horn [55,57,70], is restricted to inhibitory interneurons in this region and contributes to disinhibition in the spinal cord [70,79,80]. Because it is found on 13–15% of neurons in laminae I–II [70], we estimate that around half of the inhibitory interneurons in these laminae possess the receptor. However, we do not yet know whether it is associated with particular neurochemical types of interneuron. Some inhibitory interneurons are activated by painful stimuli [15,19,69,83,84], but little is known about the responses of cells belonging to these 4 classes. There is controversy over the extent to which nNOS-containing neurons are activated by noxious stimuli [5,18,29,31,41], and there have apparently been no studies of the responses of cells belonging to the other 3 populations.

In this study, we examined sst_2A_ expression among the different neurochemical classes and used 2 different activity-dependent markers, phosphorylation of extracellular signal–regulated kinases (ERKs) [23] and expression of Fos [22], to test their responses to noxious mechanical, thermal and chemical stimuli. The aim was to determine whether inhibitory interneurons belonging to these classes differ in their expression of sst_2A_ receptor and their responses to noxious stimuli, as this would support the idea that they represent functionally distinct populations and help to elucidate their roles in somatosensory processing.

## 2 Methods

### 2.1 Animals and tissue processing

Experiments were approved by the Ethical Review Process Applications Panel of the University of Glasgow and were performed in accordance with the UK Animals (Scientific Procedures) Act 1986.

Thirty-seven male Wistar rats (220–350 g; Harlan) were used in the study. Seven of these were deeply anaesthetized with pentobarbitone (300 mg i.p.) and perfused through the left ventricle with fixative. For 4 of the rats this contained 4% freshly depolymerized formaldehyde, while for the other 3 it contained 4% formaldehyde/0.2% glutaraldehyde. The other 30 rats were used to investigate phosphorylation of ERK or expression of Fos after noxious stimulation. Four different types of noxious stimulus (heat, pinch, or injection of capsaicin or formalin) were applied to one hind paw of these animals. For most phospho-ERK (pERK) experiments (*n* = 4–6 rats per stimulus type) the stimuli were applied while the animals were under general anaesthesia with urethane (0.4–0.8 g, i.p.), and this was maintained for 5 min after the end of the stimulus, at which point the animals were perfused with fixative (containing 4% formaldehyde). For Fos experiments (*n* = 3 rats per stimulus type), the animals were briefly anaesthetized with isoflurane (2.5–3%) while the stimulus was applied and were then allowed to recover from general anaesthesia. They were reanaesthetized with pentobarbitone and perfused with fixative (4% formaldehyde) 2 h after the stimulus. The heat stimulus involved immersion of the hind paw in water at 52°C for 20 s, while the pinch stimulus consisted of repeated pinching of folds of skin (6 each on the dorsal and ventral surface of the hind paw, applied with forceps for 5 s at each point over the course of 1 min) [42]. Chemical stimulation involved injection of 25 μL of 1% capsaicin (dissolved in 7% Tween-80, 20% ethanol, saline) [63] or 100 μL of formalin (2% formaldehyde) [8] into the plantar surface of the paw. In preliminary experiments we found that relatively few cells in the superficial dorsal horn were positive for Fos after the pinch stimulus, and this stimulus was therefore not used to investigate Fos expression. The noxious stimuli were applied while animals were anaesthetized in order to minimize discomfort. Continuous general anaesthesia with urethane was used in the pERK experiments because ERK phosphorylation peaks within 5 min after noxious stimulation, and it was therefore necessary to carry out the perfusion fixation promptly at this time [23].

In order to test for pERK expression during the second phase of the formalin response [9], 3 rats received a formalin injection in the foot while under brief isoflurane anaesthesia. They were reanaesthetized with pentobarbitone and perfused at 30 min after formalin injection. This survival time was chosen as it is near the peak of the second phase, which starts around 15 min after injection [65,78].

After perfusion fixation, midlumbar (L4–5) segments were removed from all animals and cut into 60-μm-thick sections with a Vibratome. Transverse sections were used for all parts of the study.

Sections were immersed in 50% ethanol for 30 min, and those from glutaraldehyde-fixed animals were treated with 1% sodium borohydride for 30 min (to reduce free aldehyde groups), followed by extensive rinsing. Sections were then processed for multiple-labelling immunofluorescent detection, as described below. Details of the sources and concentrations of primary antibodies are listed in Table 1. All secondary antibodies were raised in donkey and were species specific. Fluorescent secondary antibodies were conjugated to Rhodamine Red, DyLight 649 (1:100, 1:500, respectively; both from Jackson Immunoresearch) or Alexa 488 (1:500; Invitrogen). In some cases, secondary antibodies conjugated to biotin (1:500) or horseradish peroxidase (HRP; 1:1,000, both from Jackson Immunoresearch) were used. The biotinylated antibodies were revealed with avidin conjugated to Pacific Blue (1:1,000; Invitrogen) or with avidin–HRP (Sigma; 1:1,000) followed by tyramide signal amplification (TSA; tetramethylrhodamine kit; PerkinElmer Life Sciences). The HRP-labelled secondary antibodies were revealed with TSA. TSA reactions were used when 2 of the primary antibodies in an immunoreaction were raised in the same species (Fos combined with either NPY or galanin). In these cases, the initial incubation included one of these antibodies at low concentration (Table 1), and this was revealed with TSA. The sections were subsequently reacted with the other primary antibody, which was revealed with secondary antibody conjugated to a different fluorochrome [3]. For all other reactions sections were initially incubated in a cocktail containing all primary antibodies and then in a corresponding mixture of secondary antibodies. Primary antibody incubations were for 3 days and those in secondary antibodies were overnight (both at 4°C). Antibodies were diluted in PBS that contained 0.3% Triton-X100, except for reactions involving anti-sst_2A_, in which 5% normal donkey serum was included in both primary and secondary antibody solutions, and TSA reactions, in which the blocking reagent supplied by the manufacturer was used. All sections were mounted in anti-fade medium and stored at −20°C. In all cases, combinations of 3 or 4 fluorescent dyes with widely differing emission spectra (eg, Pacific blue, Alexa 488, Rhodamine Red and DyLight 649) were used.

Unless otherwise stated, sections were selected for scanning and analysis before immunofluorescence was examined. They were scanned with a Bio-Rad radiance confocal microscope (with Argon multi-line, 543 nm HeNe and 637 diode lasers) or a Zeiss LSM710 confocal (with Argon multi-line, 405 nm diode, 561 nm solid state and 633 nm HeNe lasers) through 40× oil-immersion lenses (numerical aperture 1.3) with the pin-hole set to 1 Airy unit. Overlapping fields to cover laminae I–III were scanned at 2 μm *z* separation through the full thickness of the section, except for the analysis of GABA immunoreactivity.

All quantitative analyses were carried out with Neurolucida for Confocal software (Microbrightfield). The outline of the grey matter and the border between laminae II and III were drawn for the transverse sections, and the locations of immunoreactive cells were plotted onto these outlines. The position of the lamina II/III border was determined either from dark field scans, or from the ventral border of the plexus of sst_2A_-immunoreactive dendrites [70]. Although a stereological method was not used for any of the analyses of cell counts in the *z* stacks that were obtained from the full thickness of the sections, the sampling bias towards larger neurons is likely to have been very small, as the section thickness (60 μm) was considerably larger than the cell bodies of the neurons that were being sampled.

### 2.2 Expression of sst_2A_ among different populations of interneurons

Sections from the L4 segments of 3 rats that had been fixed with 4% formaldehyde were reacted with guinea pig anti-sst_2A_, mouse monoclonal antibody NeuN [40] and rabbit antibodies against one of the following: galanin, NPY or parvalbumin. Two sections were selected from each rat for each antibody combination, and confocal scans were obtained from laminae I–III on one side for each section. Initially, only the channels corresponding to NeuN and either galanin, NPY or parvalbumin were viewed with Neurolucida, and the locations of all neurons that were galanin, NPY or parvalbumin immunoreactive were plotted. The channel corresponding to sst_2A_ was then viewed, and the presence or absence of the receptor was noted for each selected neuron.

Because nNOS is found in both inhibitory and excitatory interneurons in the rat [54], we analyzed expression of sst_2A_ by GABA-immunoreactive neurons that contained nNOS in sections from animals that had been fixed with glutaraldehyde, which provides optimal retention of GABA. Sections from L4 of 3 rats fixed with glutaraldehyde/formaldehyde were reacted with rabbit anti-GABA, sheep anti-nNOS and guinea pig anti-sst_2A_. Six or 7 sections were selected from each of the 3 animals before nNOS immunostaining was viewed, and either one or both dorsal horns in these sections were then scanned with the confocal microscope. In this way, 7 sets of scans (each corresponding to a single dorsal horn in one Vibratome section) were obtained from each of the 3 animals. Because penetration of GABA immunostaining is extremely limited in Vibratome sections [54,61], only the upper surface of the section was scanned, at 1 μm *z* separation. Initially, immunostaining for nNOS and GABA were viewed, and all nNOS^+^/GABA^+^ neurons for which part of the nucleus appeared at the upper surface of the Vibratome section were plotted. The channel corresponding to sst_2A_ was then viewed and the presence or absence of immunoreactivity was recorded for each selected neuron. We also used these sections to confirm the presence of GABA in sst_2A_ neurons. On 5 of the dorsal horns from each rat, we plotted the locations of all sst_2A_^+^ cells in laminae I–III that were present at the section surface and then examined these for the presence of GABA immunoreactivity.

### 2.3 pERK and Fos after noxious stimulation

Sections from the L4 and the rostral part of the L5 segment from animals that had received noxious heat, pinch or capsaicin injection 5 min before perfusion fixation were processed to reveal pERK together with either galanin and nNOS, or NPY and parvalbumin (guinea pig antibody). Sections from the animals that had received formalin injection under urethane anaesthesia were treated in the same way, except that sst_2A_ was also revealed in conjunction with galanin and nNOS. For each neurochemical marker, tissue from 4 rats was analysed for pERK. From each rat, 4 sections containing a relatively large number of pERK cells on the side ipsilateral to the noxious stimulus were selected and scanned with the confocal microscope. The region of the superficial dorsal horn that contained pERK cells was identified and drawn onto the outline of the dorsal horn. Expression of pERK by individual neurons was not examined at this stage [68]. All cells within this region that were immunoreactive for the marker being examined were plotted, and then the presence or absence of pERK in each cell was recorded.

Sections from the corresponding segments of the rats that had received noxious stimuli 2 h before fixation were processed to reveal Fos together with: (1) NPY and parvalbumin (guinea pig antibody), (2) nNOS and sst_2A_ or (3) galanin. The sections reacted to reveal Fos with nNOS and sst_2A_ were analysed as described above for pERK, except that 3 sections from each animal were assessed. Sections reacted for Fos, together with NPY, galanin or parvalbumin were examined to determine whether the patterns of Fos expression were similar to those observed for pERK, but they were not formally analysed.

Sections from the 3 rats that had received formalin injection under isoflurane anaesthesia (30 min survival) were reacted to reveal pERK, nNOS and sst_2A_, and 3 sections from each rat were analysed as described above.

### 2.4 Antibody characterisation

We have reported that dorsal horn immunostaining with the galanin and NPY antibodies can be abolished by pretreatment with the corresponding peptides [51,59], and staining of neurons with the galanin antibody is absent from the brains of galanin knockout mice [34]. The nNOS antibody labels a band of 155 kDa in Western blot tests of rat hypothalamus, and staining is abolished by preincubation with nNOS [17]. The rabbit and guinea pig parvalbumin antibodies were raised against mouse parvalbumin and recognize a protein band of the appropriate size on Western blot tests. The sst_2A_ antibody was raised against the C terminal 15 amino acids of the peptide sequence of the rat and mouse sst_2A_ receptor, coupled to keyhole limpet haemocyanin. Immunostaining was blocked by incubation with the peptide antigen (manufacturer’s specification). The GABA antibody was raised against GABA conjugated to porcine thyroglobulin with glutaraldehyde and demonstrated negligible cross-reactivity against other amino acids (glutamate, aspartate, glycine or taurine) [48]. The NeuN antibody was raised against cell nuclei extracted from mouse brain and found to react with a protein specific for neurons [40]. We have demonstrated that NeuN labels all neurons but does not label glial cells in the rat spinal dorsal horn [70]. The monoclonal antibody against pERK detects both ERK1 and ERK2 that are dually phosphorylated at Thr202 and Tyr204 sites, and does not cross-react with either JNK or p38 MAP kinase that are phosphorylated at the corresponding residues (manufacturer’s specification). The Fos antibody was raised against a peptide corresponding to the *N*-terminus of human Fos. Staining with both pERK and Fos antibodies in the superficial dorsal horn was restricted to somatotopically appropriate areas after noxious stimulation.

### 2.5 Statistical analysis

Kruskall-Wallis 1-way analysis of variance on ranks was used to compare pERK expression among each of the neurochemical classes of interneuron after different types of noxious stimulus and the expression of Fos in response to different noxious stimuli among the nNOS-immunoreactive cells that expressed sst_2A_. *P* values of <.05 were considered significant.

## 3 Results

### 3.1 sst_2A_ expression among neurochemical interneuron classes

The distribution of immunostaining for galanin, NPY, parvalbumin and sst_2A_ in the formaldehyde-fixed tissue was the same as that reported previously in the rat [1,28,46,51,59,64,70]. NPY-immunoreactive cells were distributed throughout lamina I–III, while galanin-immunoreactive cells were concentrated in lamina I and the outer part of lamina II (IIo) and present at much lower frequency in the inner part of lamina II (IIi) and lamina III. Parvalbumin-immunoreactive cells were largely absent from laminae I and IIo and were distributed on either side of the lamina II/III border. sst_2A_ immunoreactivity was present in a dense band that occupied laminae I and II, and at high magnification this could be seen as membrane staining that outlined the cell bodies and dendrites of some neurons [70]. Occasional sst_2A_ immunoreactive cells were seen in lamina III. In the glutaraldehyde-fixed sections, the distribution of GABA and nNOS was the same as that described previously [54], with some cells in each of laminae I–III demonstrating both types of immunoreactivity. Immunostaining with the sst_2A_ antibody had the same appearance as that seen in formaldehyde-fixed tissue.

**Fig. 1:**
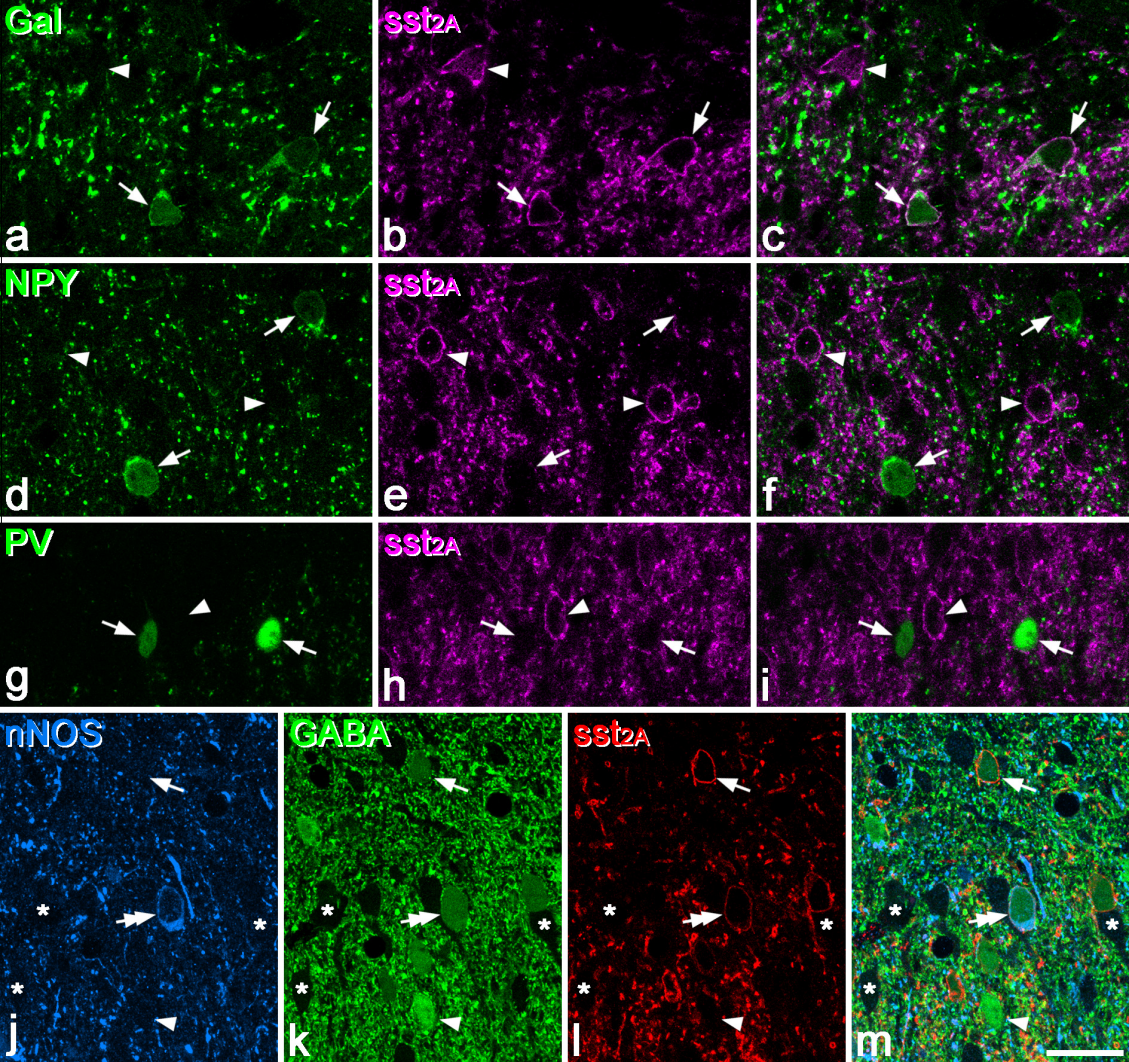
sst_2A_ expression by different neurochemical types of interneuron in lamina II in the rat. (a–c) Two galanin-immunoreactive neurons (arrows) are labelled with the sst_2A_ antibody, and a sst_2A_^+^ cell that lacks galanin is also visible (arrowhead). (d–f) Two NPY-immunoreactive cells (arrows) lack sst_2A_, which is present on other neurons (2 indicated with arrowheads). (g–i) Two parvalbumin cells are sst_2A_^−^ (arrows) and are located on either side of a sst_2A_^+^ neuron (arrowhead). (j–m) A neuron immunoreactive for both nNOS and GABA is also sst_2A_^+^ (double arrow). Two other GABA-immunoreactive neurons that lack nNOS are indicated. One of these (arrow) is sst_2A_^+^, while the other (arrowhead) is sst_2A_^−^. Several GABA^−^ cells (all of which are also sst_2A_^−^) are present in this field, and 3 of these are indicated with asterisks. All images are obtained from single confocal optical sections. Scale bar (in m) = 20 μm.

Quantitative results for this part of the study are provided in Table 2, and examples of the immunostaining are illustrated in Fig. 1. In laminae I–II, sst_2A_ was expressed by the great majority of galanin^+^ and nNOS^+^/GABA^+^ cells (97% and 93%, respectively), but only by 15% of NPY^+^ cells and 1% of PV cells. In lamina III, the receptor was found on 58% of nNOS^+^/GABA^+^ cells and a few of the galanin cells. Between 165 and 184 (mean 174) sst_2A_^+^ neurons were identified in laminae I–II in sections from the 3 rats fixed with glutaraldehyde/formaldehyde, and virtually all of these (mean 99.4%, range 99.4–99.5%) were GABA immunoreactive, consistent with our previous finding in formaldehyde-fixed tissue [70]. In the same sections the mean number of lamina III sst_2A_^+^ cells per rat was 24 (22–25), and 50% (44–56%) of these were GABA-immunoreactive. Because the restricted penetration of GABA immunostaining meant that only the superficial parts of the sections could be analysed, there will be a bias towards larger neurons (which are more likely to appear at the section surface), and it is therefore not possible to estimate proportions accurately. However, these results clearly demonstrate that virtually all sst_2A_-expressing cells in laminae I and II are GABAergic, and that the great majority of nNOS^+^/GABA^+^ cells express sst_2A_.

### 3.2 Responses of interneurons to noxious stimulation

**Fig. 2:**
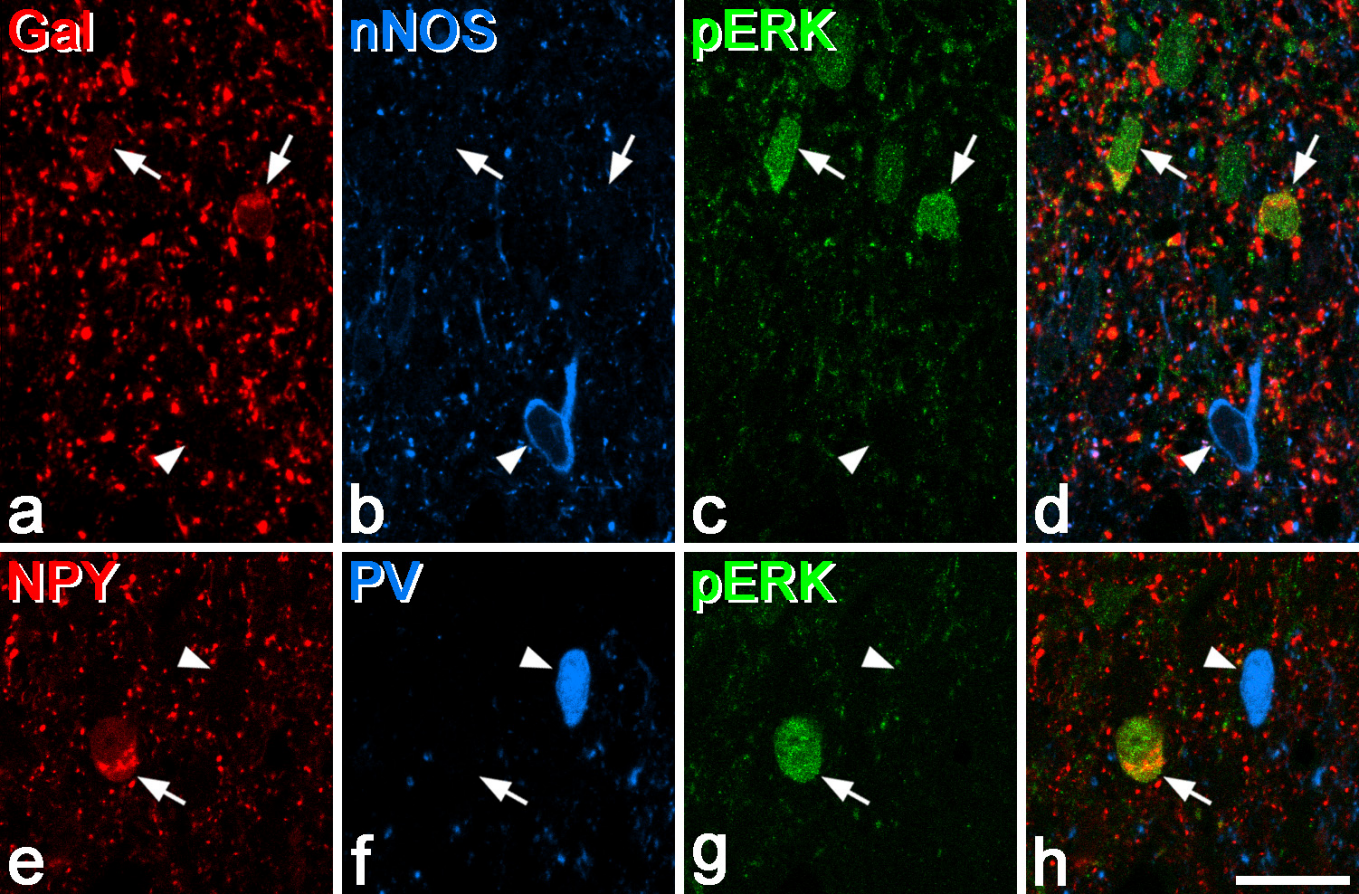
Phosphorylation of ERK in interneurons after noxious stimulation. (a–d) Part of the ipsilateral superficial dorsal horn of the L4 segment from a rat that had undergone noxious thermal stimulation of one hind paw 5 min previously. This field contains 2 neurons that are galanin-immunoreactive (arrows) and 1 that is nNOS immunoreactive (arrowhead). The 2 galanin cells contain pERK, but the nNOS cell does not. (e–h) A similar field from a rat that had received an injection of capsaicin into the ipsilateral hind paw. This contains a NPY-immunoreactive neuron that contains pERK (arrow) and a parvalbumin (PV) cell that does not (arrowhead). Both images are from single confocal optical sections. Scale bar (in h) = 20 μm.

We initially examined expression of pERK among the different neurochemical cell types in animals that had received pinch, noxious heat or capsaicin injection administered 5 min before fixation [42]. Because pERK is mainly seen in laminae I and II after these stimuli, we restricted the analysis to cells in this region (Table 3, Fig. 2). Each of these stimuli gave rise to many pERK^+^ cells in the superficial dorsal horn on the side ipsilateral to the stimulus, with a distribution similar to that reported in previous studies [23–26,42,72,82]. In all cases, virtually no pERK^+^ cells were seen on the contralateral side. A high proportion of the galanin cells in laminae I and II showed pERK in response to each of these stimuli (73%, 59% and 43%, respectively, for heat, capsaicin and pinch), while for NPY cells the corresponding values were 52%, 40% and 22%. However, very few nNOS cells (2–5%) and none of the parvalbumin cells in this region were pERK^+^ after these stimuli. Although we did not analyse lamina III, we noted that none of the parvalbumin cells in this lamina were pERK^+^ after any of the stimuli, whereas a few of the NPY and nNOS cells showed pERK, particularly in response to the pinch stimulus.

**Fig. 3:**
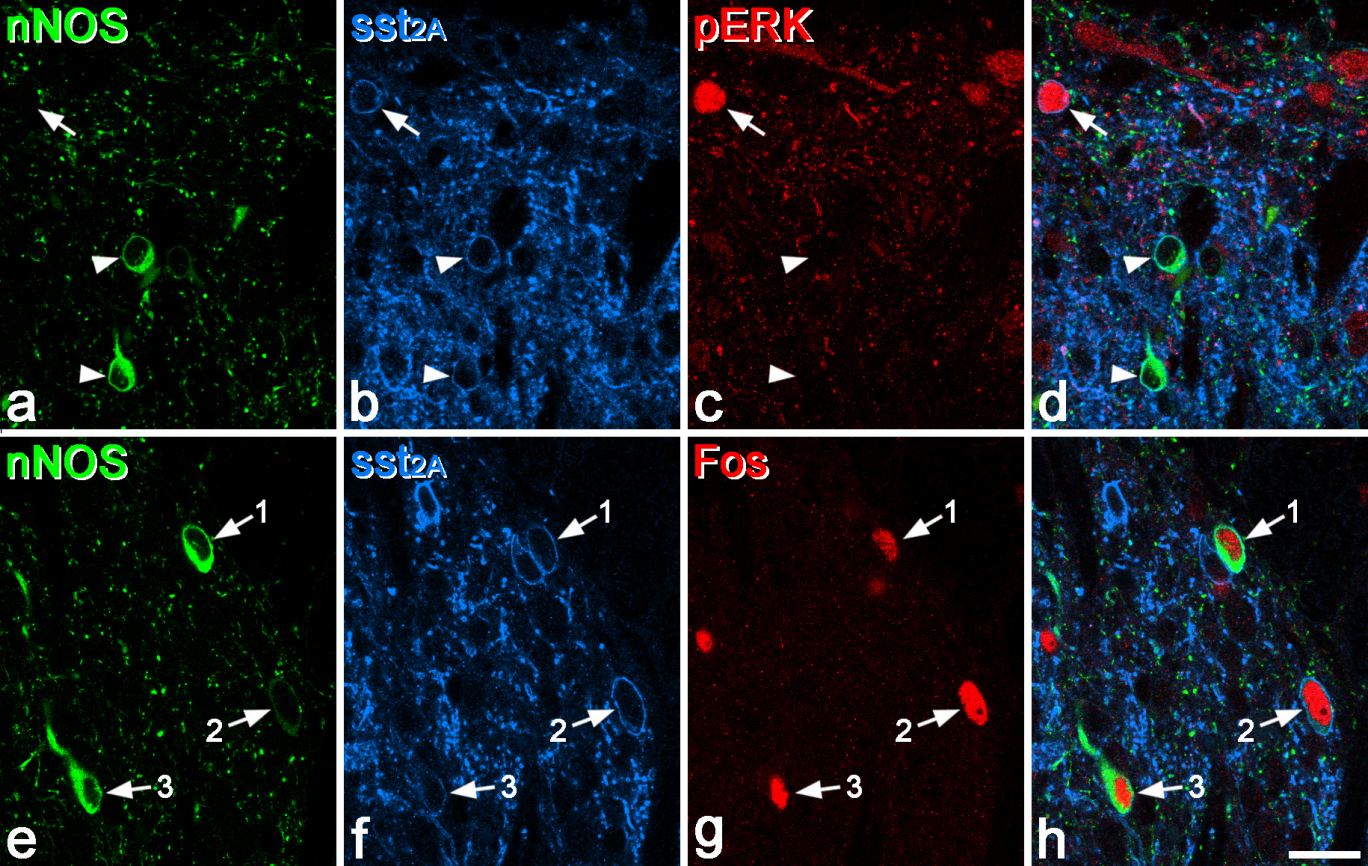
Expression of Fos but not pERK in nNOS-containing inhibitory interneurons after formalin injection. (a–d) A field from the L5 segment of a rat that had received an injection of formalin into the ipsilateral hind paw 5 min previously. Two nNOS-immunoreactive cells that express sst_2A_ (arrowheads) do not show pERK immunoreactivity, while a nearby sst_2A_ cell that lacks nNOS is pERK^+^ (arrow). (e–h) A similar field from a rat that had received an injection of formalin 2 h previously. Three nNOS^+^ cells that are sst_2A_^+^ show Fos immunoreactivity (arrows). The cells numbered 1 and 3 show strong nNOS immunostaining, whereas the cell numbered 2 is weakly labelled for nNOS. Both images are from single confocal optical sections. Scale bar (in h) = 20 μm.

Previous studies have reported that some nNOS-containing neurons in the superficial dorsal horn up-regulate Fos after subcutaneous injection of formalin [5,18,31], and we therefore also examined pERK expression in rats after injection of formalin, in particular to learn whether the nNOS cells responded specifically to this stimulus. However, although we found that pERK was present in 68% of galanin and 66% of NPY cells in laminae I–II, only 8% of the nNOS cells and none of the parvalbumin cells in this region were pERK^+^ after formalin injection (Table 3). Analysis of the responses of the 4 neurochemical populations to these 4 types of noxious stimulus with Kruskall-Wallis 1-way ANOVA on ranks demonstrated a significant difference between the populations (*P* < .001, *n* = 16 sections). Tukey’s HSD test post hoc revealed that the proportion of galanin and NPY cells with pERK was significantly higher than the proportion of either the nNOS or parvalbumin cells (*P* < .05 in each case). During the course of this study, we found that most GABAergic nNOS cells in laminae I–II expressed sst_2A_, and we therefore used the sst_2A_ antibody on the sections from formalin-injected rats that were reacted to reveal nNOS. This allowed us to identify most of the GABAergic nNOS cells in laminae I–II (ie, those that were sst_2A_^+^). Surprisingly, we found that only 1.6% (0–4%) of these showed pERK (Fig. 3a, Table 4).

We therefore tested whether these cells up-regulated Fos 2 h after formalin injection, even though they had not shown pERK 5 min after this stimulus. Although we did not analyse the behaviour of these animals, we observed that they demonstrated the expected 2-phase response, with initial licking/flinching of the injected paw that lasted for ∼5 min, followed by a prolonged second phase that started at around 15 min. Again, we used sst_2A_ antibody in order to distinguish the inhibitory nNOS interneurons. In this case, we found a very different result because the majority (69%) of nNOS^+^/sst_2A_^+^ cells in laminae I–II were Fos^+^ (Table 4, Fig. 3e–h) after formalin injection, although interestingly Fos was present in very few of the nNOS^+^/sst_2A_^−^ cells, which correspond largely to nNOS-containing excitatory interneurons. To test whether the nNOS^+^/sst_2A_^+^ cells were selectively activated by formalin, we also looked for Fos expression after noxious heat and capsaicin injection. Although only 11% of these cells showed Fos after capsaicin, the majority (73%) were Fos^+^ after noxious heat (Table 4). Kruskall-Wallis 1-way ANOVA on ranks demonstrated a significant difference between responses to the different stimuli (*P* < .001, *n* = 9 sections), while post hoc tests revealed that responses of the nNOS^+^/sst_2A_^+^ cells to capsaicin differed from those to both heat and formalin (*P* < .005, Mann-Whitney pairwise comparison with Bonferroni correction).

Although we did not quantitatively analyse Fos expression among the other neurochemical populations, this was similar to the pattern observed with pERK. Many galanin and NPY cells were Fos^+^ after formalin capsaicin or heat, while none of the parvalbumin cells showed Fos in response to any of these stimuli.

In order to determine whether the nNOS-containing inhibitory interneurons phosphorylated ERK during the second phase of the formalin test, we examined sections from rats that had received a formalin injection 30 min before perfusion fixation. Although many pERK cells were seen in laminae I–II in these animals, only 8% of the nNOS^+^/sst_2A_^+^ cells were pERK positive (Table 4).

## 4 Discussion

The main findings of this study are: (1) that in laminae I–II sst_2A_ is expressed by virtually all galanin- and nNOS-containing inhibitory interneurons, but by few NPY cells and not by parvalbumin-containing cells, (2) that ERK is phosphorylated in many galanin and NPY cells, but few nNOS cells and no parvalbumin cells after several types of noxious stimulation, and (3) that nNOS^+^ inhibitory interneurons can respond to noxious stimuli because many of them up-regulate Fos after formalin injection or noxious heat, although not after capsaicin injection.

### 4.1 Expression of the sst_2A_ receptor

**Fig. 4:**
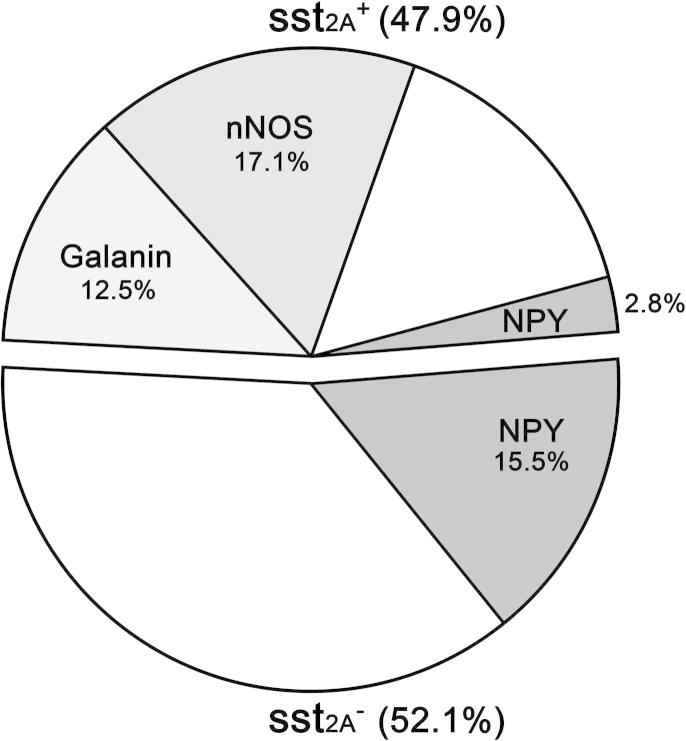
Estimated sizes of GABAergic interneuron populations in the rat superficial dorsal horn (laminae I–II). sst_2A_-expressing (sst_2A_^+^) neurons in this region are all GABAergic and make up just under half of the inhibitory interneurons. This group contains 2 large populations, which are defined by the presence of galanin or nNOS. Between them, these account for ∼60% of the sst_2A_^+^ cells. Most NPY-containing cells lack sst_2A_, but some express the receptor, and these account for ∼6% of sst_2A_^+^ cells. Percentages on the pie chart indicate the proportion of all inhibitory interneurons in laminae I–II that belong to each population. Parvalbumin-containing inhibitory interneurons are in the set of sst_2A_^−^ cells that lack NPY, but quantitative data are not available for this population. For further details, see Discussion.

We have previously reported that in the rat 24.8% and 31.3%, respectively, of neurons in laminae I and II are GABA immunoreactive [45], while the proportions that express sst_2A_ in these laminae are 13.3% and 14.6% [70]. We have also demonstrated that there are ∼7497 lamina I neurons and ∼27,465 lamina II neurons on each side in the L4 segment [43]. We therefore estimate that 29.9% of neurons in the superficial dorsal horn (laminae I–II) are GABAergic and that 14.3% express sst_2A_. Because the sst_2A_-expressing cells in this region are all GABA immunoreactive (present study and [70]), this means that they account for approximately half (47.9%) of the inhibitory interneurons in this region (Fig. 4). Most inhibitory interneurons that contained galanin or nNOS (97.4% and 93%, respectively) expressed sst_2A_, and these 2 populations are non-overlapping [64]. We have previously reported that in lamina I 26.4% of inhibitory interneurons contain galanin and 16.9% contain nNOS, while for lamina II the corresponding values are 9.9% (galanin) and 18.7% (nNOS) [54]. We therefore estimate that the sst_2A_-expressing galanin and nNOS cells account for 12.5% and 17.1%, respectively, of the inhibitory interneurons in laminae I–II (corresponding to 26.1% and 35.7% of the sst_2A_^+^ cells) (Fig. 4). NPY immunoreactivity can be detected in 23.4% of GABAergic neurons in lamina I and in 17.3% of those in lamina II [54], and these are different from the cells that express nNOS or galanin [28,64]. Only 16% of NPY-immunoreactive cells expressed sst_2A_, and we therefore estimate that the sst_2A_^+^ and sst_2A_^−^ NPY cells account for 2.8% and 15.5%, respectively, of the inhibitory interneurons in laminae I–II (Fig. 4).

nNOS is present in both inhibitory and excitatory interneurons in laminae I–II [20,54,62], and because most nNOS^+^/GABA^+^ neurons express sst_2A_, immunocytochemical detection of the receptor can be used to distinguish between these 2 cell types. This avoids the need to immunostain for GABA, which requires glutaraldehyde fixation for optimal retention. Dynorphin is also expressed by both inhibitory and excitatory interneurons in the superficial dorsal horn, with the inhibitory cells corresponding to the galanin population [3,4,53]. Immunostaining for sst_2A_ will therefore allow inhibitory dynorphin cells to be distinguished from the excitatory ones. In addition, responsiveness to somatostatin [79] can be used to identify inhibitory interneurons in patch-clamp recordings from either nNOS- or dynorphin-expressing cells.

Somatostatin administered intrathecally at physiological concentrations has a pro-nociceptive effect [58,73,74], which is thought to result from hyperpolarization of inhibitory interneurons [79]. The present results indicate that galanin- and/or nNOS-containing cells in laminae I–II are likely to contribute to this effect because they account for over half of the sst_2A_-expressing cells. The restriction of sst_2A_ to distinct populations of inhibitory interneurons provides the opportunity for exploring the functions of these cells by ablating them with a saporin conjugate, as has been used to investigate the role of other neuronal populations in the dorsal horn [27,30,35,75].

### 4.2 Responses to noxious stimulation

There were significant differences between inhibitory interneuron populations in their responses to noxious stimulation. Many galanin and NPY cells were pERK^+^ after each type of stimulus, indicating that they had been activated. In contrast, very few nNOS cells and no parvalbumin cells showed pERK in these experiments. For 3 of the stimuli (pinch, heat, capsaicin), we did not immunostain sections for sst_2A_, and the nNOS cells that were analysed will therefore have included both excitatory and inhibitory interneurons. Nonetheless, it was clear that only a very small proportion of nNOS^+^ inhibitory interneurons could have phosphorylated ERK, and this was demonstrated directly in the formalin-injected animals, in which only 2% of nNOS^+^/sst_2A_^+^ cells were pERK^+^. However, our findings with Fos indicate that a high proportion of the nNOS-containing inhibitory interneurons did respond to formalin and noxious heat, even though they did not show pERK immunoreactivity after these stimuli. General anaesthesia was maintained throughout the survival period in most pERK experiments, and this may have suppressed activation of neurons after noxious stimulation. However, this is unlikely to account for the lack of ERK phosphorylation in nNOS neurons, as we have also examined rats that survived ∼5 min after formalin injection under brief isoflurane anaesthesia, and found that they seldom showed pERK in nNOS-containing neurons (A.J. Todd and E. Polgár, unpublished data). Although ERK phosphorylation is an upstream regulator of Fos expression in superficial dorsal horn neurons [26], our results suggest that Fos can be induced in the absence of pERK, possibly through an alternative signalling pathway involving CaMKIV [14].

Previous studies have reported that some inhibitory interneurons in laminae I–II respond to noxious stimuli [19,69,83,84], but this is the first to demonstrate that these include cells belonging to the galanin and NPY populations. Several studies have investigated Fos expression among nNOS-containing dorsal horn neurons after noxious stimulation [5,18,29,31,41]. However, these have produced conflicting results. For example, Nazli et al. [41] found very few cells double labelled for nNOS and Fos after several types of noxious stimulus (mustard oil, formalin or heat), and Lee et al. reported no double-labelled cells after noxious mechanical stimulation [29]. In contrast, other studies have reported Fos in significant numbers of nNOS-containing neurons after subcutaneous injection of formalin [5,18,31]. Although it is difficult to reconcile these results, our findings clearly indicate that a high proportion of nNOS-containing inhibitory interneurons in laminae I–II can be activated by noxious stimuli.

**Fig. 5:**
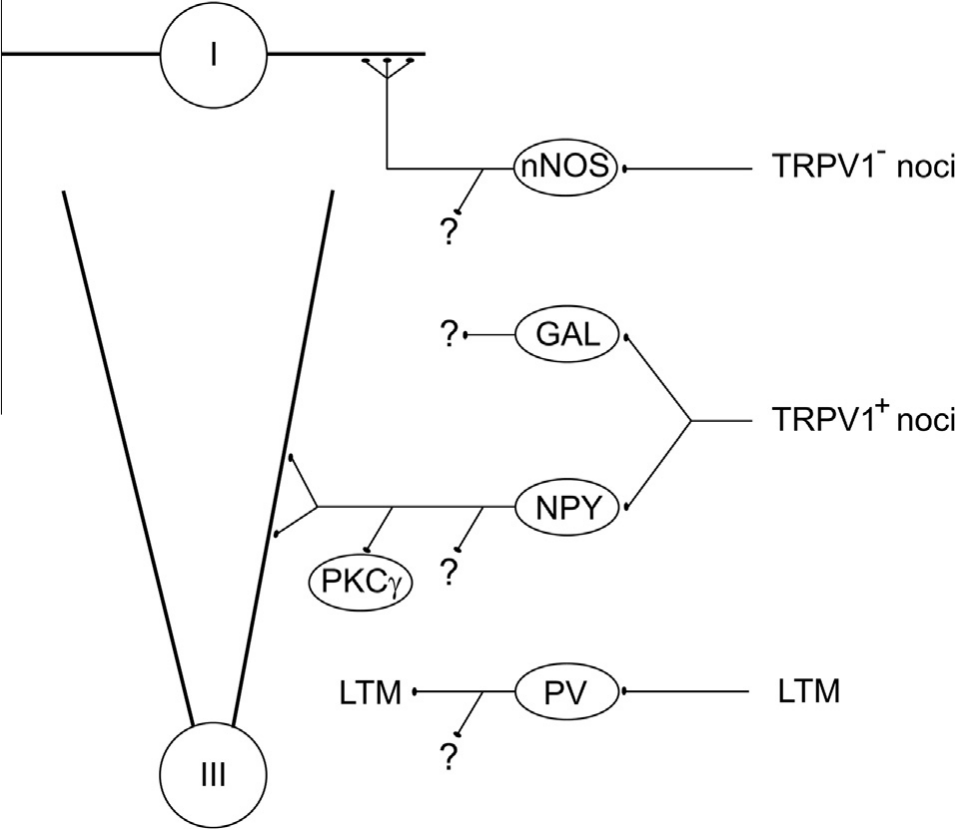
Schematic diagram summarizing the inputs to and outputs from the different interneuron populations. The results of the present study suggest that many of the galanin and NPY cells are activated by TRPV1-expressing nociceptive afferents, while the nNOS cells respond to nociceptors that lack TRPV1. These cells may also receive inputs from non-nociceptive afferents (not shown). Parvalbumin cells receive synaptic input from myelinated low-threshold mechanoreceptive (LTM) primary afferents and are probably not innervated by nociceptors. Some nNOS cells densely innervate giant lamina I projection neurons (I), while the postsynaptic targets of NPY cells include lamina III projection neurons with the NK1 receptor (III) and excitatory interneurons in lamina II that express PKCγ. Note that the NPY cells may include separate populations that innervate these 2 targets. Central boutons of low-threshold mechanoreceptive (LTM) afferents are a major target for the axons of the parvalbumin (PV) cells. Each population has other postsynaptic targets (represented by question marks), and the targets of the galanin neurons are not yet known. For further details, see Discussion.

While many nNOS^+^ inhibitory interneurons expressed Fos after heat or formalin, few did so after capsaicin injection, indicating that capsaicin is a relatively ineffective stimulus for these cells. Although many nociceptors in the rat and other species express the capsaicin receptor TRPV1 [71], a significant proportion do not [13,33,38,76]. Our results suggest that TRPV1-lacking nociceptors may preferentially innervate the nNOS cells, while TRPV1^+^ nociceptors are involved in activating galanin and NPY cells (Fig. 5).

### 4.3 Neurochemical populations of inhibitory interneurons

The finding that NPY-, galanin-, nNOS- and parvalbumin-containing inhibitory interneurons differed in receptor expression pattern and in their responses to noxious stimuli strongly suggests that these neurochemical markers reveal functionally distinct populations.

We already know that there are differences in their postsynaptic targets (Fig. 5). Two distinct targets for the axons of NPY cells have been identified: nociceptive projection neurons in lamina III that possess the neurokinin 1 receptor (NK1r), and PKCγ-expressing excitatory interneurons in lamina II [42,46,47]. These axons are thought to originate from different populations of NPY-containing interneurons [46], and it is possible that these differ in terms of laminar location and/or sst_2A_ receptor expression. Because many NPY cells respond to noxious stimulation, those innervating the lamina III projection neurons may be involved in attenuating nociceptive inputs to these cells by a mechanism involving feed-forward inhibition and thus limit the degree of pain felt after a noxious stimulus [52]. nNOS-containing GABAergic axons, which are also likely to originate from local inhibitory interneurons, selectively innervate a population of giant lamina I projection neurons that lack the NK1r [49]. Interestingly, both the giant cells [49] and the nNOS^+^ inhibitory interneurons in laminae I–II are activated by subcutaneous formalin, and nNOS cells may therefore limit the responses of the giant projection neurons after formalin injection. Nothing is apparently known about the postsynaptic targets of the galanin-containing inhibitory interneurons, except that they arborize mainly in laminae I–IIo [64]. The parvalbumin neurons largely correspond to islet cells [1,10,12], and their location in laminae III–III, together with the lack of pERK or Fos expression after various types of noxious stimulus, is compatible with the suggestion that they receive low-threshold mechanoreceptive, rather than nociceptive primary afferent input [21]. Hughes et al. have recently demonstrated that axons of the parvalbumin cells form axo-axonic synapses onto myelinated low-threshold mechanoreceptors, and they are therefore likely to generate the surround inhibition necessary for maintaining tactile acuity [21]. It is important to note that each of these neurochemical classes of inhibitory interneuron may be further subdivided into distinct populations. It is also likely that there are additional functional populations still to be identified among the inhibitory interneurons that lack galanin, nNOS, NPY or parvalbumin, and that these will include cells responding to noxious stimulation.

Ross et al. reported that loss of inhibitory interneurons in mice lacking the transcription factor Bhlhb5 leads to increased itching [50]. We have recently found that *Bhlhb5*^−/−^ mice exhibited substantial depletion of both galanin- and nNOS-containing inhibitory interneurons, but not of NPY or parvalbumin cells (A.J. Todd, E. Polgár and S.E. Ross, unpublished observations). Because many of the galanin and nNOS cells are activated by noxious stimuli, one or both of these populations may contribute to scratch-mediated inhibition of itch.

## Conflict of interest statement

The authors report no conflict of interest.

## Figures and Tables

**Table 1:** Antibodies used.

Antibody	Species	Dilution	Source
Galanin	Rabbit	1:1,000 1:20,000^a^	Bachem
NPY	Rabbit	1:1,000 1:100,000^a^	Bachem
nNOS	Sheep	1:2,000	P.C. Emson
Parvalbumin	Rabbit	1:500	M. Watanabe
Parvalbumin	Guinea pig	1:2,500	M. Watanabe
sst_2A_	Guinea pig	1:2,000	Gramsch Laboratories
GABA	Rabbit	1:5,000	D.V. Pow
NeuN	Mouse	1:500	Millipore
pERK	Mouse	1:500	Santa Cruz Biotechnology
Fos	Rabbit	1:5,000 1:40,000^a^	Santa Cruz Biotechnology

a Used in combination with the TSA (tyramide signal amplification) method.

**Table 2:** Expression of sst_2A_ by different neurochemical types of interneuron in the dorsal horn.^a^

	Laminae I + II		Lamina III	
	No. counted	% sst_2A_	No. counted	% sst_2A_
Galanin	82.3 (69–96)	97.4 (95–100)	3.3 (1–5)	8.3 (0–25)
NPY	107.3 (86–118)	15.5 (11.6–21.2)	35.7 (25–43)	2.1 (0–4)
Parvalbumin	24 (21–26)	1.3 (0–3.8)	50.3 (42–59)	0
nNOS/GABA	35.7 (31–42)	93 (90.3–97.6)	15.3 (14–17)	57.6 (35.3–73.3)

a Data are presented as mean (range) for 3 animals.

**Table 3:** pERK in different neurochemical types of neuron in laminae I–II.^a^

	Pinch		Heat		Capsaicin		Formalin	
	No. of cells	% pERK	No. of cells	% pERK	No. of cells	% pERK	No. of cells	% pERK
Galanin	39.5 (25–50)	43.1 (33.3–60)	82.5 (61–95)	73.4 (69.7–78.7)	67 (59–81)	59 (48.5–70.4)	62.8 (50–70)	67.7 (57.1–76)
NPY	60.8 (54–67)	21.7 (18.5–23.4)	80 (53–108)	52.3 (48.8–58.3)	88.8 (82–102)	39.8 (37.3–45.1)	89 (73–101)	66.4 (64.9–68.5)
nNOS	150.8 (105–197)	2.4 (1.1–3.4)	191 (151–240)	5.1 (2.5–7.9)	157 (88–207)	4.1 (2.8–5.7)	126 (92–161)	8.3 (4.3–12.4)
Parvalbumin	8.8 (6–12)	0	14.8 (10–18)	0	9.8 (7–15)	0	7.8 (5–9)	0

a Data are presented as mean (range) for 4 animals.

**Table 4:** pERK and Fos expression by nNOS^+^/sst_2A_^+^ cells in laminae I–II after noxious stimulation.^a^

Stimulus	No. of cells	% pERK or Fos
Formalin 5-min survival pERK (urethane) (*n* = 4)	32 (24–42)	1.6 (0–4)
Formalin 30-min survival pERK (isoflurane) (*n* = 3)	29 (26–34)	8 (7.4–8.8)
Formalin 2-h survival Fos (*n* = 3)	35 (26–41)	68.7 (63.4–76.9)
Heat 2-h survival Fos (*n* = 3)	29.7 (27–34)	73.4 (55.6–82.4)
Capsaicin 2-h survival Fos (*n* = 3)	37.3 (34–43)	11.3 (5.7–16.3)

a Data are presented as mean (range) for 3 or 4 animals. All noxious stimuli for Fos experiments were administered under brief isoflurane anaesthesia.
